# Rescue of Recent Virulent and Avirulent Field Strains of Bluetongue Virus by Reverse Genetics

**DOI:** 10.1371/journal.pone.0030540

**Published:** 2012-02-17

**Authors:** René G. P. van Gennip, Sandra G. P. van de Water, Christiaan A. Potgieter, Isabel M. Wright, Daniel Veldman, Piet A. van Rijn

**Affiliations:** 1 Department of Virology, Central Veterinary Institute of Wageningen UR (CVI), Lelystad, The Netherlands; 2 Onderstepoort Veterinary Institute, Onderstepoort, South Africa; Institute for Animal Health, United Kingdom

## Abstract

Since 1998, Bluetongue virus (BTV)-serotypes 1, 2, 4, 9, and 16 have invaded European countries around the Mediterranean Basin. In 2006, a huge BT-outbreak started after incursion of BTV-serotype 8 (BTV8) in North-Western Europe. More recently, BTV6 and BTV11 were reported in North-Western Europe in 2008. These latter strains are closely related to live-attenuated vaccine, whereas BTV8 is virulent and can induce severe disease in ruminants, including cattle. In addition, Toggenburg orbivirus (TOV) was detected in 2008 in Swiss goats, which was recognized as a new serotype of BTV (BTV25). The (re-)emergency of known and unknown BTV-serotypes needs a rapid response to supply effective vaccines, and research to study this phenomenon. Recently, orbivirus research achieved an important breakthrough by the establishment of reverse genetics for BTV1. Here, reverse genetics for two recent BTV strains representing virulent BTV8 and avirulent BTV6 was developed. For this purpose, extensive sequencing of full-genomes was performed, resulting in the consensus sequences of BTV8/net07 and BTV6/net08. The recovery of ‘synthetic BTV’, respectively rgBTV8 and rgBTV6, completely from T7-derived RNA transcripts was confirmed by silent mutations by which these ‘synthetic BTVs’ could be genetically distinguished from wild type BTV, respectively wtBTV6 and wtBTV8. The *in vitro* and *in vivo* properties of rgBTV6 or rgBTV8 were comparable to the properties of their parent strains. The asymptomatic or avirulent properties of rgBTV6 and the virulence of rgBTV8 were confirmed by experimental infection of sheep. Reverse genetics of the vaccine-related BTV6 provides a perfect start to develop new generations of BT-vaccines. Reverse genetics of the virulent BTV8 will accelerate research on the special features of BTV8, like transmission by species of *Culicoides* in a moderate climate, transplacental transmission, and pathogenesis in cattle.

## Introduction

Bluetongue virus (BTV) belongs to the family *Reoviridae*, genus *Orbivirus* [1]. BTV-transmission between ruminants, including cattle, sheep, and goats, occurs in majority by bites of species of *Culicoides*. Bluetongue (BT) is listed as a ‘notifiable disease’ by the Office International des Epizooties (OIE) [2] causing severe hemorrhagic disease with fever, lameness, coronitis, swelling of the head (particularly the lips and tongue) and death. There are 24 BTV-serotypes recognized as defined by cross neutralization assays.

The genome of BTV consists of ten linear double-stranded RNA genome segments Seg-1 to Seg-10 encoding structural proteins VP1 to VP7, and nonstructural proteins, NS1, NS2 and NS3/NS3a, for reviews see Roy *et al.* [3], [4]. The virus particle composes three shells of proteins, the inner shell consists of VP3 encoded by Seg-3, the middle shell consists of VP7 encoded by Seg-7, and the outer shell is formed by VP2 (Seg-2) and VP5 (Seg-6). The BTV-particle further contains three enzymatic proteins, VP1 (Seg-1), VP4 (Seg-4) and VP6 (Seg-9), and one copy of each of the ten genome segments Seg1 to Seg-10 in the inner shell. The nonstructural proteins NS1, NS2, and NS3/3a encoded by Seg-5, Seg-8, and Seg-10, respectively, are not part of the BTV particle.

Since 1998, BTV-serotypes 1, 2, 4, 9, and 16 have invaded European countries around the Mediterranean Basin. In 2006, a huge BT-outbreak started after incursion of BTV8/net06 (IAH collection nr. BTV-8 NET2006/04) [5] in N-W Europe [6]. More recently, BTV6 (BTV6/net08) and BTV11 were reported in N-W Europe in 2008 [7], [8], [9]. Both BTV-strains are closely related to live-attenuated vaccine strains (IAH collection nr. BTV-6 NET2008/05) [9], [10] Further, a new BTV-member, Toggenburg orbivirus (TOV), was detected in goats in Switzerland in 2008, and is proposed to be the 25^th^ serotype of BTV [11].

These incursions and the re-emergency of BTV-serotypes in Europe are addressing the need for safe and effective vaccines, but also for research on the recent development of Bluetongue in areas with a moderate climate. Spread of Bluetongue is restricted to the expansion of the competent vector, specific species of *Culicoides*. BTV8/net06 must be transmitted by endemic European *Culicoides* spp. [12], [13], [14], as the common vector *C.* imicola was not found in the affected area. Further, Bluetongue is generally believed to be a ‘sheep disease’, but BTV8/net06 is causing significant clinical signs in cattle [15]. Finally, BTV8/net06 is passing the placenta of pregnant heifers and ewes, although this is restricted to BT-vaccines or cell-adapted BTV-strains [16]. It was therefore suggested that BTV8/net06 should be related to a BT-vaccine or a laboratory strain of BTV8. To study the specific properties of BTV8/net06, a method to genetically manipulate BTV is required. For BTV8/net06 and BTV6/net08, a genetic modification system based on the uptake of a plasmid-derived T7-transcript was published [17]. However, this system is not efficient, laborious, and needs positive selection to remove wild type virus. Recently reverse genetics was developed based on T7 RNA-polymerase driven run-off transcripts for BTV1 [18]. This reverse genetics system was successfully used to genetically modify BTV [19], [20], [21]. The reverse genetics system offers many advantages, like the efficient rescue of virus with fully defined genomes, recovery of mutant viruses with delayed growth characteristics, and tailor-made BTV-reassortants. Here the rescue of completely ‘synthetic’ virulent BTV8 (rgBTV8), and completely ‘synthetic’ avirulent BTV6 (rgBTV6) is described. Both ‘synthetic’ BTVs are studied in sheep in order to confirm their properties compared to their wild type strains, wtBTV8 and wtBTV6.

## Materials and Methods

### Cell lines and virus

BSR cells (a clone of BHK-21 cells; gift from Polly Roy, [22]) were cultured in Dulbecco's modified Eagle's medium (DMEM; Invitrogen) containing 5% fetal bovine serum (FBS) and 1% Penicillin/Streptomycin and Fungizone.

*C. variipennis* (KC) cells (gift from Linda McHolland, [23]) were grown in modified Schneider's Drosophila medium with 15% heat inactivated fetal bovine serum, 4.5 mU/ml bovine insulin, 6 mg/L reduced glutathione, 30 mg/L L-asparagine, 2 ml 7.5% sodium bicarbonate and 2 mM L-glutamine to the medium of which the pH is adjusted to 6.7–6.8 with 1N hydrochloric acid [23].

BTV8/net07 was isolated from the Holstein Frisian cow NL441689187 from Bavel, the Netherlands, which was sampled for export purposes on July 24th, 2007 [24]. Isolation from EDTA-blood was performed on eggs (e1) and subsequent three passages on BHK-21 cells (e1/bhk3) or subsequent three passages on KC cells (e1/kc3). BTV6/net08 was isolated from the Holstein Frisian cow NL415834681 (24th October 2008) from Heeten, the Netherlands [7]. Isolation from EDTA-blood was performed on eggs and subsequent three passages on BHK-21 cells and once on BSR cells.

All virus stocks were obtained by infection of BSR cells at low multiplicity of infection (MOI) and harvested after 100% cytopathic effect (CPE) was observed. Virus titers were determined by endpoint dilution and expressed as plaque forming units per ml. Viral stocks were stored at −80°C.

### Sequencing of BTV8/net07 and BTV6/net08

Embryonated egg-isolated BTV8/net07/e1, cell-culture derived BTV8/net07/e1/bhk3, BTV8/net07/e1/kc3 and BTV6/net08/e1/bhk3/bsr2 were send to C.A. Potgieter at Onderstepoort Veterinary Institute (South Africa). BTV8/net07 and BTV6/net08 genome segments Seg-1 to Seg-10 were sequenced after use of an improved strategy for sequence-independent amplification of segmented dsRNA viral genomes [25] and pyrophosphate-based 454 (Roche GS20/FLX) sequencing at the Inqaba Biotec company (SA). Sequence analysis was essentially done as described [25] using Lasergene8 from DNASTAR. Files containing the sequence information, quality values and flowgrams (sff files) were loaded into the Seqman 8 programme of the Lasergene software.

Contig sequences of BTV8 strains were checked manually and compared to sequences of BTV-8nt(2006/04) [5] with genbank accession numbers AM498051–AM498060) and to sequences of BTV8/Neth2006 submitted by A.C. Potgieter ([25] with genbank accession numbers FJ183374–FJ183383 for Seg-1 to Seg-10.

Consensus sequences of BTV8 directly isolated from EDTA-blood were determined after RNA-isolation using the TRIZOL method, and reverse transcription and amplification by a one-tube system (Qiagen one-step RT-PCR kit). Overlapping DNA amplicons were sequenced using BigDye® Terminator v1.1 Cycle Sequencing Kit in a ABI PRISM® 3130 Genetic Analyzer Applied Biosystems.

Contig sequences of BTV6/net08 were checked manually and compared to sequences of BTV6/Net2008/05 [10] with genbank accession numbers QG506472–QG506481.

### Construction of T7 plasmids with cDNA-inserts derived from BTV genome segments

**Figure 1:**
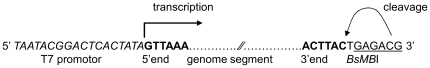
Schematic overview of plasmids containing a full-length BTV genome segment. A full-length BTV genome segment flanked by a T7 promoter and an unique site for a restriction enzyme (here *Bsm*BI is indicated as an example), which defines the BTV 3′end sequence during transcription (depicted from Boyce et al., [18]. The nucleotides of the ultimate 5′- and 3′-ends of the BTV genome segment are presented in bold symbols. The sequence of the T7 promoter is italicized, and that of the unique site is underlined. The positions of the start of transcription and digestion by restriction enzymes for run-off transcription are indicated by arrows.

cDNA of **g**enome segments were synthesized by Genscript Corporation (Piscataway, NJ). Genome segments Seg-1 to Seg-10 of BTV1 were based on the sequences as submitted to Genbank with accession numbers FJ969719–FJ969728 [18]. Genome segments Seg-1 to Seg-10 of BTV8/net07 and of BTV6/net08 were based on the consensus sequences described in this paper. cDNAs were cloned in pUC-derivatives, pUC18, 19 or 57, or pJET1.2 under control of the T7 RNA-polymerase promoter and a recognition site for a restriction enzyme at the 3′-terminus for defined run-off transcription (Figure 1, depicted from Boyce et al., [18]). Plasmids were maintained in *E.coli* DH5α, and were purified using the QIAfilter Plasmid Midi Kit (Qiagen).

### *In vitro* synthesis of BTV-transcripts

Plasmid DNA was digested at the 3′-terminus with the respective restriction enzyme, and was purified by standard procedures. One µg digested plasmid DNA was used for *in vitro* RNA run-off transcription with 5′-cap analogue using the MESSAGE mMACHINE T7 Ultra Kit (Ambion). In this reaction, a ratio of 4∶1 of anti-reverse cap analogue to rGTP was used. Synthesized RNA was purified by use of MEGAclear columns (Ambion) according to the manufacturer's instructions, and eluted RNA was stored at −80°C.

### Transfection of T7-derived RNA transcripts to rescue ‘synthetic’ BTV1, BTV6 and BTV8

Monolayers of 10^5^ BSR cells per 2 cm^2^ were transfected with equimolar amounts of RNA of BTV segments encoding VP1, VP3, VP4, NS1, VP6, NS2. In total, 600 ng RNA was transfected using 1 µg lipofectamine™ 2000 (1∶2.5; 1 mg/ml Invitrogen) in Opti-MEM® I Reduced Serum Medium according to manufacturer's conditions. Eighteen to twenty hours post transfection, monolayers were transfected again with 600 ng equimolar amounts of ten RNA segments. At 4 hrs post transfection, the transfection mix was replaced with 1 ml DMEM supplemented with 5% FBS and 1% of Penicillin/Streptomycin/Fungizone. Supernatants were harvested from monolayers developing cytopathogenic effect (CPE) at 48 hrs after the second transfection. BTV-specific CPE was confirmed by immunostaining of fixed monolayers with monoclonal antibody (Mab) produced by ATCC-CRL-1875 directed against VP7 according to standard procedures. In case of monolayers without visible CPE, duplicate wells were passed 1∶5 to rescue virus from less efficiently transfected monolayers. After incubation of three days, monolayers were screened for CPE and VP7-expression as described above.

### Characterization of rescued BTVs

Viral RNA of virus stocks was isolated from 200 µl of supernatant with the High Pure Viral RNA kit (Roche). Genome segments Seg-2 and Seg-6 were identified by amplification of (parts of) the genome segments with serotype-specific primers, which are listed in Table 1. Template RNA (6 µl) was denaturated at 94°C for 3 min and immediately cooled on ice. A one-step RT-PCR kit (Qiagen) was used to reversely transcribe RNA and amplification of cDNA in a RT-PCR containing both primers. The reaction mix contained 10 µl of 5xQIAGEN one-step RT-PCR buffer, 2 µl of dNTP mix, 0.6 µM of each primer and 2 µl of the enzyme mix (containing RT and PCR reaction enzymes). RNase-free water was added to a total volume of 44 µl. Six microliters of denatured RNA was added to the mix. The RNA was reversely transcribed at 45°C for 30 min. This was followed by an activation step at 94°C for 15 min. Forty amplification cycles were then carried out (94°C for 1 min, 45°C for 1 min and 72°C for 2 min), followed by a terminal extension step at 72°C for 10 min. The cDNA products were analyzed by 0.9% agarose gel electrophoresis and visualized under UV light after staining with ethidium bromide.

**Table 1:** List of forward and reverse primers used for amplification of (parts of) genome segment 2 of BTV6 and BTV8.

primer^a^	sequence	expected amplicon size (bp)
BTV8S2f	GTTAAAATAGCGTCGCGATG	964
BTV8S2r6	GATGTTGGTACGCTCGTT	
BTV6S2f8	TGATCCACGACTCAATGGAC	791
BTV-S2-2R	TTTCTGATGCGATCAATG	

a All primers were supplied by Eurogentec b.v., Maastricht, Netherlands.

For Seg-10, the in-house developed serogroup-specific diagnostic PCR-test was used [15]. Sequencing of amplicons was carried out to discriminate between different Seg-10 sequences by using the BigDye® Terminator v3.1 Cycle Sequencing Kit in a ABI PRISM® 3130 Genetic Analyzer (both supplied by Applied Biosystems, Foster City, IA, USA).

### Growth curves and plaque morphology of ‘synthetic’ BTV6 and 8 (rgBTV6 and rgBTV8)

Confluent BSR-monolayers in M24-well plates were infected in duplicate at a multiplicity of infection (moi) of 0.1. After attachment to cells for 1.5 h at 37°C, the medium was removed and refreshed with 1 ml of DMEM with 5% FBS, 1% Penicillin/Streptomycin/Fungizone and incubation was continued. At 24, 48 and 72 h post infection (hpi), samples of the supernatants were harvested and stored at −80°C. Virus titers were determined by plaque-titration on BSR cells. Therefore, BSR cells were infected with tenfold dilutions of samples, and grown for 48 h under EMEM overlay medium (containing 1% methylcellulose). Infectious centers (hereafter referred to as plaques) were detected by immunostaining with a VP7-specific Mab ATCC-CRL-1875. Virus titers were expressed as pfu/ml.

To study plaque size, BSR monolayers were infected by tenfold dilutions of indicated viruses, and grown for two days under overlay medium (EMEM complete with 1% methylcellulose). Monolayers were fixed with methanol/aceton (1∶1) and immunostained with Mab ATCC-CRL-1875. Plaques in appropriate dilutions were compared.

### Experimental infection with rgBTV6 and rgBTV8

All experiments with live animals were performed under the guidelines of the European Community (86/609) and were approved by the Committee on the Ethics of Animal Experiments of the Central Veterinary Institute (Permit Number: 2011-003). Sixteen female Blessumer sheep of 6–24 months old and free of BTV and BTV-antibodies were commercially sourced from the same flock of a Dutch farm. The sheep were randomly allocated to four groups of four animals. On day 0 (0 dpi), a total of 1 ml of 10^5^ TCID_50_/ml of wtBTV8 or rgBTV8 was administered subcutaneously to four sheep per virus between the shoulder blades left and right from the spinal cord. Four sheep were intramuscularly injected in the neck with 1 ml of 10^5^ TCID_50_/ml rgBTV6. The fourth group served as control group. EDTA-blood samples were collected daily during the first week after inoculation and every other day until the end of the trial at 21 dpi. Serum samples were collected more frequently in the second week post inoculation. Samples were tested by the in-house PCR-test for BTV [15] for the detection of BTV RNA in EDTA-blood and by blocking ELISA (ID-VET) for the detection of BTV-specific antibodies in serum. Body temperature was recorded daily, and fever was defined as the average temperature plus two times the standard deviation. Clinical signs were recorded daily according to the clinical score table for BTV8 animal trials (Table S1).

Clinical signs were quantified following challenge by an adapted clinical reaction index (CRI) as described by Huismans [26]. A maximum score of 12 was given to the cumulative total of fever readings (a) as described above from days 3 to 14 post inoculation (dpi), a clinical score according the clinical score table and normalized to a maximum score of 12 (b). An additional 4 points were added to the sum of a and b if death occurred within 14 dpi.

## Results

### Recovery of synthetic BTV1

Rescue of infectious BTV1 from all ten *in vitro* synthesized RNAs has been previously demonstrated [18]. With the aim of the development of reverse genetics for BTV we started reproducing this technique for BTV1. Synthesized and purified run-off transcripts were used to rescue BTV1 according to the double transfection protocol [21] with small modifications. Briefly, 2 cm^2^ of BSR-monolayers were transfected with in total 600 ng equimolar amounts of RNA. Virus was directly harvested from supernatant of transfected monolayers, or after passage of the transfected monolayer in case no CPE was seen. In general, CPE could be seen as early as 24 h post transfection, however, supernatants were harvested at 48 h post transfection to increase virus recovery. Monolayers were immunostained with VP7-specific Mabs in order to check specificity of CPE. BTV1 was efficiently rescued with a virus titer of about 10^6^ pfu/ml. Supernatant of transfected monolayers was used to prepare a stock of ‘synthetic’ BTV1 by one passage on BSR cells.

### Recovery of rgBTV8

cDNAs from genome segments Seg-1 to Seg-10 of BTV8/net06 were synthesized based on genbank accession numbers AM498051–AM498060 [5]. However, new sequences became available for this strain [25] showing differences with the mentioned accession numbers. In order to start with a well-documented strain of BTV8 we decided to extensively sequence the BTV8-isolate of the first reported case in 2007 [24]. Consensus sequences were determined of BTV8/net07/e1, BTV8/net07/e1/bhk3, and BTV8/net07e1/kc3 by deep sequencing. Consensus sequences were compared, and only in BTV8/net07/e1/kc3 two nucleotide differences were found. In Seg-1 at nucleotide position 3277 C→T leads to amino acid change A→V and in Seg-2 position 1215A→G leads to amino acid change R→G. The consensus sequences determined by deep sequencing of BTV8/net07/e1 and BTV8/net07/e1/bhk3 were the same as that of blood-derived BTV8/net07 which was determined by conventional RTPCR and sequencing and compared to sequences of BTV8/Neth2006 (Table 2).

**Table 2:** Sequence differences between strain BTV8/2007 and BTV8/Neth2006.

RNA segment	nucleotide position^a^	BTV8Neth2006/04^b^	BTV8Neth2006^c^	BTV8Neth2007^d^	amino acid change^e^	Remarks
Seg-1	1128	t	c	c	-	
	1226	g	a	g	-	
	1454	g	a	a	-	
	1491	c	g	g	Q493E	
	2051	c	c	t	-	
	2534	c	c	t	-	
	2634	a	a	g	T875A	
	3701	c	c	t	-	
	3939	c	a	a	-	3′UTR
Seg-2	242	a	a	g	-	
	443	g	g	a	-	
	470	c	c	t	-	
	1007	c	c	a	-	
	2084	g	a	g	-	
Seg-3	38	g	a	a	-	
	62	t	a	a	-	
	113	c	c	t	-	
	1328	t	t	c	-	
	2471	c	t	t	-	
Seg-4	620	g	g	a	-	
	1026	a	a	t	M340L	
	1324	t	g	g	F439G	
	1960	a	g	g	-	3′UTR
	1969	c	a	a	-	3′UTR
	1971	t	g	g	-	3′UTR
Seg-5	1432	c	t	c	-	
	1639	c	c	t	-	
	1768	t	-	t	-	3′UTR
Seg-6	19	c	t	t	-	5′UTR
	144	a	a	g	-	
	546	a	a	g	-	
	1365	c	c	t	-	
	1383	a	g	g	-	
Seg-8	194	g	a	a	D59N	
	781	g	a	a	-	
	1112	g	-	g		3′UTR
Seg-9	11	-	ta	-		5′UTR
	22–24	cta	gct	gct	L3A	
	141	g	g	a	A43T	
	256–258	gac	aga	aga	D81R	
	413	t	t	c	A133V	
	863	g	a	a	-	
	1020–1022	tct	ctc	ctc	-	3′UTR
	1024	c	t	t	-	3′UTR
	1041–1044	acat	taca	taca	-	3′UTR

a Nucleotide position numbering from strain BTV8/Neth2007 (genbank accession numbers GQ506451–GQ506460).

b Strain BTV8/Neth2006/04 [5] has been isolated on KC cells and passaged one time on BHK21 cells before analysis. The sequences from the genome segments correspond to genbank accession numbers AM498051–AM498060 for Seg-1 to Seg-10.

c Strain BTV8/Neth2006 [25] is the same strain as BTV8/Neth2006/04 but has been passaged 2 times extra on BHK21 cells. The sequences from the genome segments correspond to genbank accession numbers FJ183374–FJ183383 for Seg-1 to Seg-10.

d BTV8/net07/e1/bhk3 (described in this paper) was isolated on eggs and was further passaged 3 times on BHK21 cells before analysis. This sequence matches completely to sequences determined by Maan et al (2010). See also footnote a.

e Amino acid position numbering from strain BTV8/Neth2007.

No differences were found when the sequences were compared to BTV8/Net2007/01 [10] with genbank accession numbers GQ506451–GQ506460. However, fifty-five nucleotide differences were found which were different either with BTV8/2006/04 [5] or BTV8/2006 [25]. These differences resulted in 9 amino acid changes in 6 different proteins and 11 changes in 5- or 3′UTR's.

The consensus sequence of BTV8/net07 was used to generate cDNAs encoding the correct amino acid sequence and contain the correct 5′- and 3′-UTR. Therefore, differences in the 5′- and 3′-UTR and changes resulting in amino acid mutations in the originally derived cDNAs (based on BTV8/Net2006) were corrected by standard procedures with respect to the consensus sequence from BTV8/net07. Consequently, 16 silent mutations remained in cDNAs used for rescue of BTV8/net07.

T7 run-off transcripts from these corrected cDNAs were transfected according to the modified double transfection protocol. CPE appeared between 24–48 h after the second transfection. In general, CPE seemed to be slightly delayed compared to BTV1.

**Figure 2:**
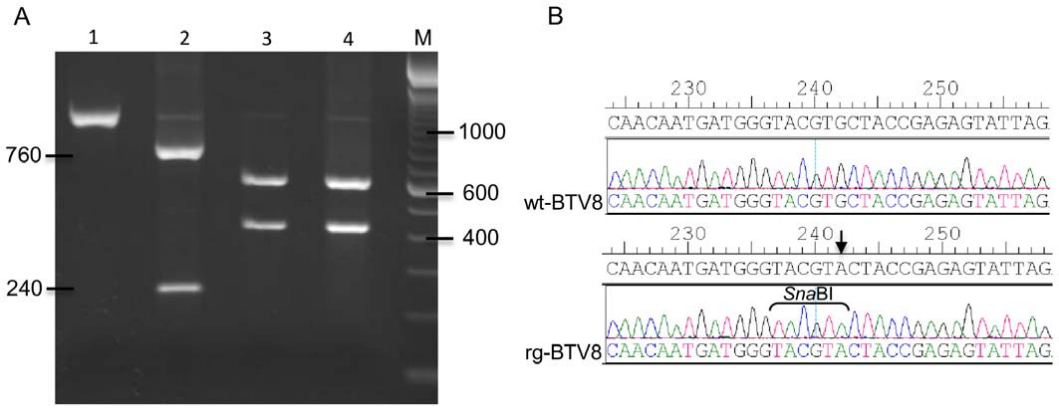
Characterization of rgBTV8 containing a marker mutation. (A) *Sna*BI or *Xho*I digestion of Seg-2 RT-PCR products. *Sna*BI- or *Xho*I-digested RT-PCR products amplified from isolated RNA of strains rg- and wtBTV8 (passage 3) with primers btv8-S2f and btv8-S2r6 were separated on 1,5% agarose gel. Lanes 1–2 *Sna*BI-digestion: lane 1, wtBTV8; lane 2, rgBTV8. Lanes 3–4 *Xho*I -digestion: lane 3, wtBTV8; lane 4, rgBTV8. Marker sizes (in bp) are indicated on the right (TrackIt™ 100 bp DNA ladder Invitrogen). Sizes of *Sna*BI-digested fragments are indicated on the left (in bp). (B) Sequence electropherograms of the Seg-2 RT-PCR products of wt- and rgBTV8 including the Seg-2 *Sna*BI mutation in rgBTV8 at position 242 (nucleotide position in segment indicated above sequences).

To confirm the rescue of ‘synthetic’ BTV8 (rgBTV8), a straightforward method was used to distinguish rgBTV8 from wild type BTV8 (wtBTV8). A part of Seg-2 encompassing a *Sna*BI recognition site that is specific for rgBTV8 (position 242) was amplified by RT-PCR using primers btv8-S2f and btv8-S2r6. Digestion by *Sna*BI of amplicons derived from wtBTV8 or rgBTV8 resulted in fragments of 1000 bp or 760 and 240 bp respectively (Fig. 2A, lane 1 wtBTV8 vs lane 2 rgBTV8). This demonstrated that a *Sna*BI site was uniquely present in rgBTV8. Successful digestion by *Xho*I confirmed the specificity, and comparability of both amplicons (lane 3–4). The amplicons were also sequenced using the btv8-S2f primer to indeed confirm the introduction of this distinguishable sequence (Fig. 2B).

The genetic stability of rgBTV8 was studied by sequencing the entire genome, except for the ultimate sequences at the 5′- and 3′ends. RNA was isolated from the supernatant of the third passage, sequenced by the described method, and compared to sequences of the cDNAs used for rescue. Except for one silent mutation in Seg-3 at nucleotide position 260, the determined sequences of Seg-1 to Seg-10 were confirmed, including all silent mutations. In conclusion, ‘synthetic’ BTV8 (rgBTV8) was recovered, is genetically stable, and confirmed by the presence of silent mutations in the RNA segments of rgBTV8. These silent mutations, 17 in total, and divided over 6 genome segments, are unique and distinguish rgBTV8 from wild type BTV8 (wtBTV8).

### Recovery of rgBTV6

Sequences of Seg1 to Seg-10 derived from BTV6/net08 e1/bhk3/bsr1 were 100% identical with those of BTV6/Net2008/05 (accession numbers QG506472–QG506481) isolated by one passage on KC cells and three passages on BHK-21 cells. These consensus sequences were used for cDNA-synthesis. Two mutated *Lgu*I recognition sites of which one was changed to an *Nla*IV-site were introduced in the cDNA of Seg-2 without affecting the encoded amino acid sequence.

**Figure 3:**
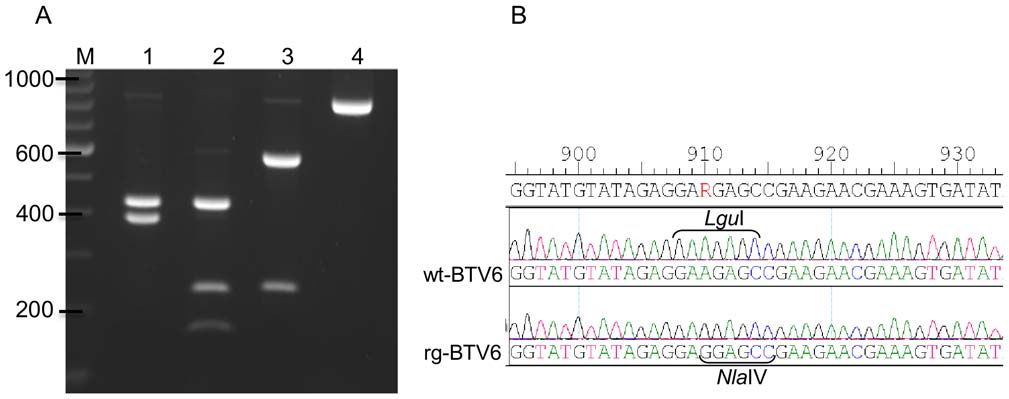
Characterization of rgBTV6 containing a marker mutation. (A) *Nla*IV or *Lgu*I digestion of Seg-2 RT-PCR products. *Nla*IV or *Lgu*I-digested RT-PCR products amplified from isolated RNA of strains rg- and wtBTV6 (passage 3) with primers btv6-S2f8 and btv6-S2-2R were separated on 1,5% agarose gel. Lanes 1–2 *Nla*IV-digestion: lane 1, wtBTV6; lane 2, rgBTV6. Lanes 3–4 *Lgu*I-digestion: lane 3, wtBTV6; lane 4, rgBTV6. Marker sizes (in bp) are indicated on the left (TrackIt™ 100 bp DNA ladder Invitrogen). (B) Sequence electropherograms of the Seg-2 RT-PCR products of wt- and rgBTV6 including the Seg-2 *Lgu*I mutation in rgBTV6 at position 910 (nucleotide position in segment indicated above sequences).

T7 RNA-transcripts from the digested BTV6/net08 plasmids were transfected according to the optimized protocol as described. CPE appeared between 1–2 days post the second transfection. The presence of rgBTV6 was confirmed by digestion with *Nla*IV instead of the *Lgu*I at position 910 in Seg-2. Therefore, a part of Seg-2 segments of rgBTV6 and wtBTV6, encompassing the respective region were partially amplified by RT-PCR using primers btv6-S2f8 and btv6-S2-2R. Digestion of these amplicons demonstrated that an extra *Nla*IV site was introduced in rgBTV6 compared to wtBTV6 (Fig. 3A, lanes 1 and 2). Digestion with *Lgu*I further confirmed that this recognition site was present in wtBTV8 and mutated in rgBTV8 (Fig. 3A, lanes 3–4). Sequences of the respective amplicons indeed confirmed the introduction of the distinghuishable, but silent, mutation (Fig. 3B). In conclusion, ‘synthetic’ BTV6 (rgBTV6) was recovered, and could be differentially identified by the presence of silent mutations.

### Characterization of rescued ‘synthetic’ BTVs rgBTV6 and rgBTV8

**Figure 4:**
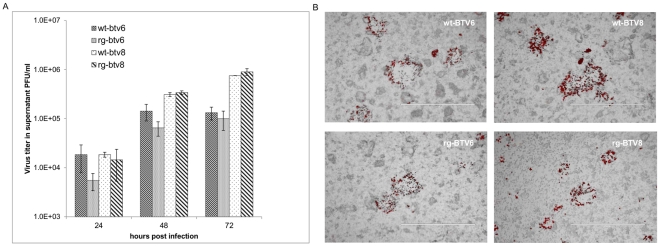
Growth curve and plaque size of viruses rgBTV6, wtBTV6, rgBTV8 and wtBTV8. (A) BSR monolayers were infected in duplicate by indicated viruses with 0.1 moi. At 24, 48 and 72 hours post infection, samples of 1 ml supernatant were taken. The virus titer in collected samples were determined by plaque-titration, expressed as pfu/ml, and plotted at a logarithmic scale. The bars represent the averages and standard errors from two replicates. (B) BSR monolayers were infected by indicated viruses in tenfold dilutions and grown for 2 days under overlay medium (EMEM complete with 1% methylcellulose). Plaques in appropriate comparable dilutions are displayed.

To compare rgBTVs with wtBTVs, growth curves in BSR cells were determined. Therefore, virus titers were measured at 24, 48, and 72 hpi (Fig. 4A). Although at 24 hpi both rgBTV6 and rgBTV8 grew to slightly lower virus titers (5,5.10^3^ and 1,45.10^4^ pfu/ml, respectively) than wtBTV6 or wtBTV8 (1,85.10^4^ and 1,85.10^4^ pfu/ml, respectively), the virus titer at 72 hpi was similar (Fig. 4A.,1,0.10^5^ and 1,33.10^5^ pfu/ml for BTV6, and 9,0. 10^5^ and 7,5.10^5^ pfu/ml for BTV8).

Plaque morphology of the ‘synthetic’ viruses, rgBTV6 and rgBTV8, grown under overlay medium for two days was similar to that of the wtBTVs (Fig. 4B). In conclusion, rgBTV6 and rgBTV8 derived from ten in vitro synthesized T7 transcripts are indistinguishable from wtBTV6 respectively wtBTV8 with respect to growth characteristics in BSR cells. Furthermore, the introduced silent mutations rgBTV6 (two mutations in Seg-2) and in rgBTV8 (16 mutations in genome segments Seg-2, Seg-3, Seg-4, Seg-5, Seg-6 and Seg-8) did not influence these growth characteristics.

### Virulence of rescued ‘synthetic’ BTVs rgBTV6 and rgBTV8

**Figure 5:**
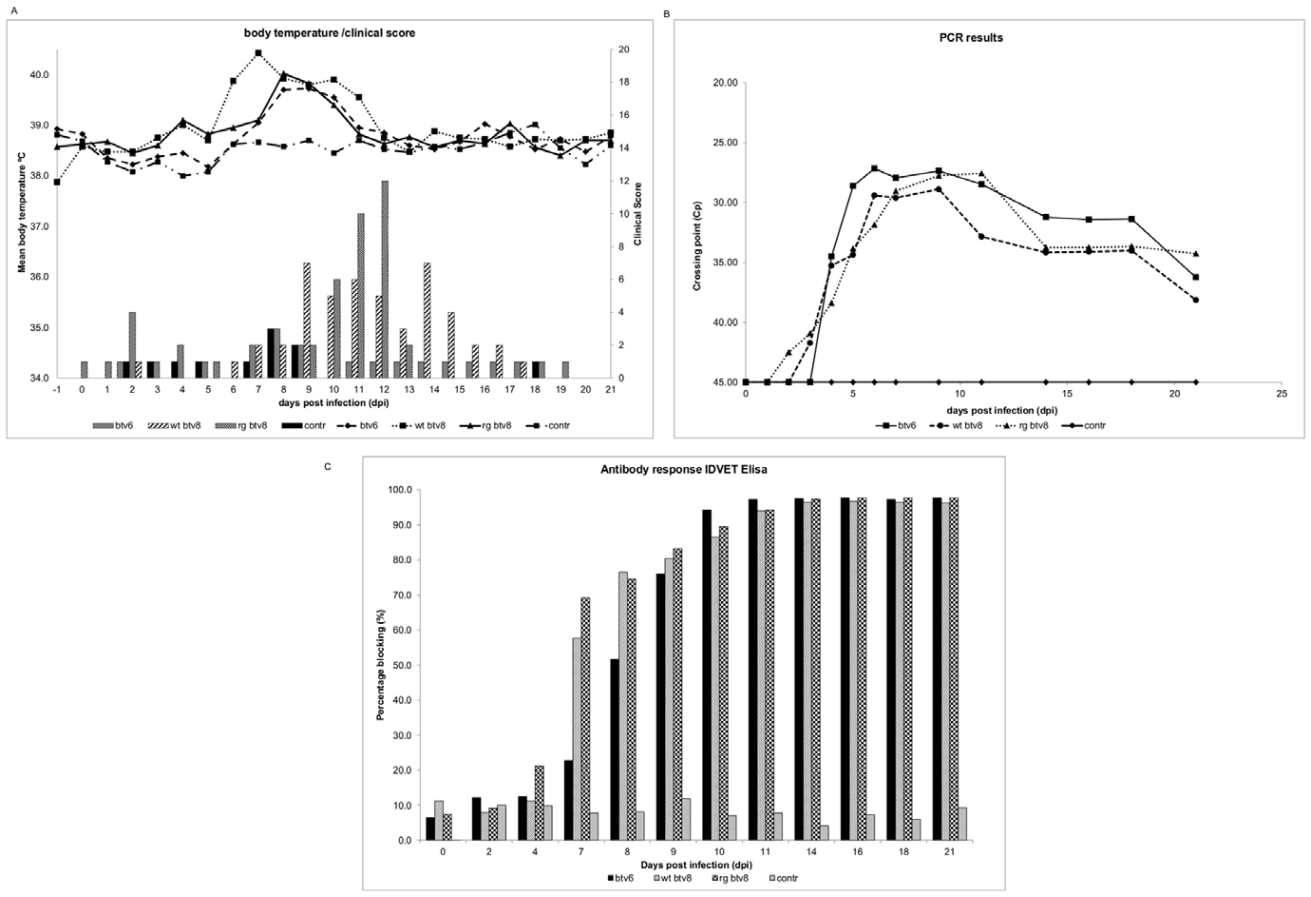
Experimental infection of sheep with viruses rgBTV6, rgBTV8 and wtBTV8. Four sheep per group were infected with 10^5^ TCID50/ml rgBTV6 (i.m.) or 10^5^ TCID50/ml rg- or wtBTV8 (s.c.). (A) body temperatures (mean values per group; lines) and clinical scores (per group; bars) were recorded daily. (B) the presence BTV genomic RNA was determined through a real-time Seg-10 RTPCR (mean values per group). (C) the presence of BTV specific antibodies in serum samples was determined with a BTV-VP7 blocking Elisa (ID-VET) and the mean blocking percentage per group was displayed as 100-value (sample).

All sheep of the control group remained negative by PCR, and ELISA. None of the sheep in the control group developed fever and only very mild clinical signs were observed, like nasal/ocular discharge and upper airway distress. Sheep infected (i.m.) with rgBTV6 developed very mild signs of BT (Fig. 5A), like loss of appetite for one day, mild ocular lacrimation and upper airway distress and/or nasal discharge for more than one day. All infected sheep developed fever between days 8 and 11 post infection (p.i.) and were PCR-positive for BTV from five dpi onwards the end (21 dpi) (Fig. 5B). All infected sheep seroconverted in the blocking ELISA (Fig. 5C) from 8 dpi onwards.

Sheep infected (s.c.) with wtBTV8 developed mild to moderate clinical signs of BT (Fig. 5A). Except for one sheep, which had no clinical signs at all, all sheep scored mild clinical signs as mentioned for rgBTV6. More severe clinical signs were observed from 9 dpi onwards. Two of the animals were listless, showed local oedema and red mucous in the mouth for several days. All sheep developed fever between 6 and 11 dpi. All sheep became PCR-positive between day 3–6 dpi and remained PCR-positive until the end of the trial (21 dpi) (Fig. 5B). Sheep seroconverted by ELISA from 7 dpi onwards (Fig. 5C).

Sheep infected (s.c.) with rgBTV8 developed similar clinical signs as the wtBTV8-infected sheep (Fig. 5A), except for one sheep, which showed no clinical signs at all. One sheep died at 8 dpi. This sheep also showed moderate clinical signs like apathy, salivation, red mucous in the mouth and conjunctivitis. All sheep in this group developed fever between 8–10 dpi. All sheep were tested PCR-positive from 3–9 dpi onwards (Fig. 5B), and seroconverted for VP7 antibodies from 7 dpi onwards (Fig. 5C). The sheep in both groups, infected with wtBTV8 and rgBTV8 which did not show clinical signs, developed fever, became PCR-positive and seroconverted, which confirmed successful infection.

The adapted clinical reaction index (CRI) was determined for each sheep and averaged for the different groups to further quantify clinical signs in groups (in particular wt- and rgBTV8). For the control group the CRI (mean ± SE) was 1.1±0.4, for the rgBTV6 group 3.9±0.8, for the wtBTV8 group 8.7±2.2 and for rgBTV8 group the CRI was 7.9±3.3. This showed a significant difference (p<0.01) between the control group and the inoculated groups. There was also a significant difference in the CRI for rgBTV6 and both BTV8-groups, although the variation in the BTV8-groups was high. There was no significant difference in CRI between the wtBTV8- and the rgBTV8 -group.

Blood samples were used to determine the sequence of parts of Seg-2, as described above, and a part of Seg-3 for only rgBTV8 and wtBTV8. For all viruses, the determined sequences matched the sequence of the BTV administered to the sheep.

## Discussion

After comparison of available sequences of the European BTV8, many differences were found between BTV8/Net2006/04 (KC1/BHK1; [5]), BTV8/Net2006/04 (KC1/BHK3; [25]) and BTV8/Net2007/01 (KC1/BHK1; [10]). Since a correct sequence is crucial to establish reverse genetics, it was decided to restart with the well-documented BTV8 isolate of 2007.

For this purpose, the complete sequences were determined by deep sequencing of three different strains originating from the same BTV8-isolate from the same cow. In addition, to exclude possible artefacts by the isolation procedure or the method of sequencing, RNA of BTV8 was directly isolated from EDTA-blood and almost completely sequenced by conventional sequencing. Except for three passages in KC cells (BTV8/net07/e1/kc3), the sequences of BTV8/net07/e1 and BTV8/net07/e1/bhk3 completely match the sequence of the original blood-isolate. Indeed, BTV8/net07/e1/bhk3 induces similar clinical signs as the blood-isolate (manuscript in preparation). In contrast, two differences were found after three passages in KC cells, which could be involved in adaptation to this cell line. Previously derived cDNAs were corrected with respect to this consensus sequences, but the remaining 16 nucleotide differences did not result in change of encoded amino acids or mutations in UTR-sequences. Consequently, rescued virus harbours genetic markers in six genome segments to identify the presence of synthetic BTV8 (rgBTV8). It has been shown for Sindbis- and poliovirus that silent mutations can affect the attenuation [27], [28]. Similarly, remaining genetic differences could change the characteristics of rgBTV8 compared to wtBTV8, but no differences were observed in growth kinetics, plaque morphology and virulence. It can therefore be concluded that these remaining silent mutations had no effect on the characteristics of BTV8. Furthermore, all silent mutations remained present in rgBTV indicating that there is no strong positive selection pressure for the original genetic background of BTV8/net07. Finally, one extra silent mutation was found in Seg-3 at nucleotide position 260.

Except for Seg-7 and Seg-10 (no mutations), and Seg-2 and Seg-3 (silent mutations), obvious (nonsilent) variations were found in all other genome segments of BTV8/Neth2006 compared to the consensus sequences of BTV8/Neth2007 and strains derived from BTV8/net07, as listed in Table 2. Remarkably, all these genome segments (Seg-1, -4, -5, -6, -8, and -9) showed variation in the sequences of UTR-sequences, and some of these are located in the highly conserved “ACTTAC” sequence [29]. Of course, these differences could be involved in translation and/or replication of the respective genome segment. In addition, genome segments (Seg-1, -4, -8, and -9) showed variation in the encoded amino acid sequences. For Seg-1, the amino acid (aa) difference at position 493 (Q493E) is located between the N-terminal domain (NTD: residues 1–373) and the Polymerase domain (PD; residues 582–882) of VP1. The other aa difference T875A is located within the PD, but is located outside the conserved sequence motifs A–D [3] at the end of the PD in the thumb region. For Seg-4, aa difference M340L is located in the 2'OMT domain (residues 155–377) of VP4. The aa difference F439G is located in the second region of the N7MTase domain (residues 110–154 and 378–509). For Seg-8, aa difference D59N is located in the N-terminal region of NS2 (1–115) which is involved in formation of viral inclusion bodies, binding of ssRNAs, and replication. Finally, most aa differences were found in Seg-9. These aa differences, M3L, A43T, D81R and A133V are not located in any of the conserved motifs that are characteristic for RNA helicases of the DExH type [30]. Summarizing, the cause and effect of these observed variations in several determined sequences of closely related strains of BTV8/Neth06 are unknown.

Most of the clinical signs associated to Bluetongue were observed after experimental infection of sheep with BTV8, and were consistent with clinical signs reported most frequently in the field in 2006 [31]. The most prominent of these were fever, apathy, erosions of the oral mucosa, salivation, local oedema and some problems with feet. To minimize interbreed susceptibility to BTV, sheep from the same flock were used. However, the severity of the clinical signs varied within a group, with moderate clinical signs in two of the sheep, but relatively mild (one sheep) to no clinical signs in the other sheep.

For the BTV6/net08 the consensus sequence was determined of BTV6/net08/e1/bhk3/bsr2 and was 100% identical to that of BTV6/Neth2008/05 (E1/KC1/BHK1) [10]. Virus rgBTV6 was synthesized based on this consensus sequence, except for two silent mutations in Seg-2, which were introduced for differentiation between rgBTV6 and wtBTV6. In fact, BTV6/net08 is likely a reassortant of two live-attenuated vaccines, since 9 out of 10 genome segments are almost identical (99.7 to 100%) to these of live-attenuated vaccine for serotype 6 (MLV6), whereas the variable genome segment Seg-10 showed the greatest identity (98.4%) to that of MLV2 (RSAvvv2/02) [10]. Indeed, BTV6/net08 hasn't shown obvious clinical signs in the field and after experimental infection [8], [32]. Furthermore, transmission of BTV6/net08 was very low in N-W European conditions resulting in a natural decline, and finally BTV6/net08 disappeared in the winter of 2008/2009 without control measures were undertaken. Similar growth characteristics of wtBTV6 and rgBTV6 were observed (Fig. 4), and the absence of virulence of rgBTV6 was similar to previous observations after experimental infection of sheep, the most susceptible and sensitive ruminant for Bluetongue. This vaccine-related BTV6/net08 caused fever in all sheep, but showed very mild clinical signs in sheep compared to rgBTV8/net07.

Reverse genetics was developed for two recent field isolates of BTV, the virulent strain of BTV8 and the vaccine-related strain of BTV6. These BTV-isolates differ in many aspects, like serotype, virulence, pathogenesis in sheep and cattle, and transmission by *Culicoides* species which are endemic for N-W European countries with a moderate climate. Reverse genetics can be used to generate desired reassortants of these different viruses. Reassortment is a natural process of viruses with a segmented genome. Reassortants have also been described for BTV for a long time (reviewed in [33]). However, these ‘natural’ reassortants will arise by positive selection, even in cell culture. Previously, we have used this process by transfection of synthetic RNA in a BTV-infected cell, followed by screening for the desired ‘synthetic’ resassortant meaning with the synthetic genome segment [17]. However, this method was not very efficient and limits the possibilities. Reverse genetics can increase this method by formation of sets of desired genome segments for rescue of viable BTV. Even less fit virus will be rescued due to the absence of negative selection. In order to investigate this application, we have rescued multiple reassortants of BTV1 and BTV8 during the process of developing reverse genetics (Table 3). These reassortants were all viable indicating the flexibility of combination of sets of ten genome segments of BTV1 and BTV8.

**Table 3:** Schematic overview of ‘synthetic’ reassortants between rgBTV1 and rgBTV8.

Virus No	Virus Name^a^	Virus Segments^b^									
		VP1	VP2	VP3	VP4	NS1	VP5	VP7	NS2	VP6	NS3
BTV104	BTV1/8 (5, 8)					x			x		
BTV105	BTV1/8 (2, 5, 8)		x			x			x		
BTV106	BTV1/8 (4, 5, 8)				x	x			x		
BTV107	BTV1/8 (5, 6, 8)					x	x		x		
BTV108	BTV1/8 (5, 7, 8)					x		x	x		
BTV109	BTV1/8 (5, 8, 9)					x			x	x	
BTV110	BTV1/8 (5, 8, 10)					x			x		x

a The segments derived from BTV8 are shown between brackets.

b Virus segments coding for indicated proteins from BTV8 are shown by an “x”.

However the ‘synthetic’ BTVs, rgBTV6 and BTV8, are genotypically distinguishable from their ‘ancestor’ viruses, BTV6/net08 and BTV8/net06, these ‘synthetic BTVs’ are phenotypically indistinguishable from their ‘ancestors’ as studied for several *in vitro* parameters and virulence in sheep. Other aspects, including transplacental transmission and replication in the vector, are not studied in detail yet. Reverse genetics as genetic modification system, here demonstrated by the introduction of many silent mutations, will be a powerful tool for in-depth investigations by site-directed mutagenesis of viral genes involved in these intriguing phenomena of bluetongue virus and other orbiviruses.

## Supporting Information

Table S1 **Clinical score table BTV animal trial.** The clinical signs described in this table were based on findings in the field [31] as well as experimental data [15] and scored daily depending on severity from 0–3 points during 3–15 days post inoculation.
(DOCX)
