# Supplementary material for: Rescue of Recent Virulent and Avirulent Field Strains of Bluetongue Virus by Reverse Genetics
Source: PLoS One. 2012 Feb 17;7(2):e30540. doi: 10.1371/journal.pone.0030540 (PMC3281837; doi:10.1371/journal.pone.0030540)
Supplement: Table S1 — Clinical score table BTV animal trial. The clinical signs described in this table were based on findings in the field [31] as well as experimental data [15] and scored daily depending on severity from 0–3 points during 3–15 days post inoculation. (DOCX) [file pone.0030540.s001.docx]

**Table S1.** Clinical score table BTV animal trial

|  | **absent (0)** | **mild (1)** | **moderate (2)** | **severe (3)** |
| --- | --- | --- | --- | --- |
| **Depression** | Responsive  (sheep is alert and responsive to environmental stimuli) | Sheep is listless | Apathy, sheep react only if stimulated, separating from the flock | Prostration, not responsive to stimuli |
| **Salivation** | No secretion | Lower jaw wet from saliva | Sheep is actively secreting | Hypersalivation |
| **Facial oedema** | Absence of oedema | Local oedema | Multiple oedemas localized in different regions | Marked facial oedema, “bottle jaw” (submandibular oedema) |
| **Lower airway distress** | Normal respiratory rate*  (≤ 40 breath/min) | Slightly increased respiratory rate* (41-60) and/or abnormal breathing sounds | Increased respiratory rate*  (61-100) and/or clear abdominal breathing | Strongly increased respiratory rate* (>100) and/or rapid abdominal breathing and/or mouth breathing/abnormal breathing sound (froth) |
| **Nasal discharge**  **Upper airway distress** | Absence of ocular/nasal clinical signs | Intermittent serous (clear) to mucoid (gray and cloudy) discharge/lacrimation | Persistent mucoid (gray and cloudy) to purulent (thick and yellowish/green) discharge | Purulent (thick and yellowish/ green) to bloody discharge |
|  | Normal breathing | Rare coughing/sneezing (only during physical activity) | Frequent coughing/sneezing  (also at rest) | Frequent coughing/sneezing at rest with prolonged episodes |
| **Muco-cutaneous and oral lesions**  **Ocular discharge** | No lesions | Hyperemia and inflammation labial mucosa/gums, blisters appear on oral mucosa | Petechial and ecchymotic hemorrhages in the oral mucosa/oral erosions | Confluent erosions oral mucosa/ulcers |
|  | Absence of ocular clinical signs | Lacrimation/ red mucous membranes | Discharge/conjunctivitis | Cataractal, white haze, conjunctivitis |
| **Dermal and hoof coronet lesions** | No lesions/normal derma | Reddening of skin above coronary band, sheep doesn’t show any difficulty of movements | Coronitis, warm lower limbs and hooves (body mass is moved to other legs) | Severe lameness, stiff gait,  hunched appearance/sheep may kneel/lying down/crippled |
